# Enhancing Diagnostic Infrastructure Through Innovation-Driven Technological Capacity in Healthcare

**DOI:** 10.3390/healthcare13243328

**Published:** 2025-12-18

**Authors:** Nicoleta Mihaela Doran

**Affiliations:** Department of Finance, Banking and Economic Analysis, Faculty of Economics and Business Administration, University of Craiova, 200585 Craiova, Romania; nicoleta.doran@edu.ucv.ro

**Keywords:** innovation scoreboard, medical imaging technology, technological infrastructure, healthcare modernization

## Abstract

**Background:** This study examines how national innovation performance shapes the diffusion of advanced diagnostic technologies across European healthcare systems. Strengthening technological capacity through innovation is increasingly essential for resilient and efficient health services. The analysis quantifies the influence of innovation capacity on the availability of medical imaging technologies in 26 EU Member States between 2018 and 2024. **Methods:** A balanced panel dataset was assembled from Eurostat, the European Innovation Scoreboard, and World Bank indicators. Dynamic relationships between innovation performance and the adoption of CT, MRI, gamma cameras, and PET scanners were estimated using a two-step approach combining General-to-Specific (GETS) outlier detection with Robust Least Squares regression to address heterogeneity and specification uncertainty. **Results:** Higher innovation scores significantly increase the diffusion of R&D-intensive technologies such as MRI and PET, while CT availability shows limited responsiveness due to market maturity. Public health expenditure supports frontier technologies when strategically targeted, whereas GDP growth has no significant effect. Population size consistently enhances technological capacity through scale and system-integration effects. **Conclusions:** The findings show that innovation ecosystems, rather than economic growth alone, drive the modernization of diagnostic infrastructure in the EU. Integrating innovation metrics into health-technology assessments offers a more accurate basis for designing innovation-oriented investment policies in European healthcare.

## 1. Introduction

In the last decade, European health-care systems have been undergoing a profound transformation shaped by demographic change, global health crises, technological progress, and the pursuit of sustainability. The growing complexity of health needs, coupled with the acceleration of digital technologies, has made innovation not merely an option but a prerequisite for maintaining resilience and equity in health provision [1]. Health systems are expected to deliver better outcomes with limited resources, while simultaneously adapting to the demands of an ageing population and the implications of chronic disease. These pressures have intensified calls for innovation as a strategic mechanism for improving efficiency, accessibility and quality of care across Europe [2,3].

Innovation in health care is a multifaceted process that extends beyond the introduction of new technologies. It encompasses organisational reforms, novel financing approaches, integrated service delivery, and digital transformation. According to the European Observatory on Health Systems and Policies, innovation must be seen as an adaptive capability that allows health systems to evolve in response to societal expectations and technological opportunities [4]. This understanding moves the discourse away from innovation as a discrete event and toward innovation as a systemic property—one that shapes governance, infrastructure and workforce development. The capacity of health systems to absorb, scale, and sustain innovations is therefore a central concern for European policy frameworks.

The European Commission recognises that research and innovation represent the foundation for both competitiveness and social wellbeing. Within frameworks such as Horizon Europe and EU4Health, the emphasis is placed on fostering collaborative environments that connect research, technology and health-policy communities to accelerate the translation of scientific knowledge into health-system improvements [5,6]. These programmes reflect a shift from isolated research efforts to an integrated policy agenda that treats innovation as a public good contributing simultaneously to economic recovery and societal resilience. By linking research performance to health outcomes, the European Union seeks to ensure that advances in technology and knowledge directly support the sustainability and responsiveness of health-care systems.

The COVID-19 pandemic has served as a pivotal reminder of the need for adaptable and innovation-driven health governance. Rapid vaccine development, data sharing, and digital health tools illustrated the transformative potential of science and technology when embedded in a supportive ecosystem. At the same time, the pandemic exposed structural weaknesses: uneven access to digital infrastructure, fragmented research capacities, and insufficient coordination across sectors [7]. As health systems seek to rebuild, attention is increasingly directed toward strengthening innovation ecosystems that connect universities, industries, policymakers and care providers. Such integration is essential not only for addressing immediate crises but also for long-term preparedness against demographic, environmental and epidemiological challenges.

Technological advancement plays a key role in this broader vision. The World Health Organization emphasises that the future of health systems will depend on the ability to harness innovation ethically and inclusively—ensuring that digitalisation, artificial intelligence and new medical technologies contribute to equity rather than deepen disparities [8]. Yet, despite Europe’s strong research base, the diffusion of innovation across health systems remains uneven. Differences in institutional capacity, regulatory frameworks and investment priorities can determine whether scientific progress translates into practical improvements for patients and providers. Addressing these gaps requires a dual focus: strengthening national innovation systems while fostering cross-border collaboration and knowledge transfer.

Recent European policy discourse has increasingly associated innovation with the concept of technological capacity—the structural readiness of a health system to adopt, implement and sustain new solutions [8]. Technological capacity includes not only the availability of equipment and infrastructure, but also the human capital, governance mechanisms and financial instruments that enable innovation to scale. Building smarter health systems therefore implies cultivating environments where innovation thrives through coherent policy, strategic investment and evidence-informed management. As articulated in the Health at a Glance: Europe 2024 report, countries that invest in innovation governance and research performance are better positioned to enhance the efficiency and resilience of their health systems [1].

In this context, analysing the relationship between innovation and health-system transformation has become a crucial line of inquiry. While the evidence base continues to expand, consensus is emerging that innovation serves as both an outcome and a driver of system performance—shaping the capacity to deliver sustainable, high-quality care in a rapidly changing environment. Understanding how innovation ecosystems function within European health contexts provides valuable insights into the conditions that enable technological progress to translate into improved outcomes and stronger institutional resilience.

## 2. Literature Review

Literature on innovation in and around health systems reveals recurring tensions between technological advancement, prioritization, equity, and sustainability. Within pharmaceutical services, recent discourse cautions that enthusiasm for first-in-class products may outpace system capacity, budgets, and evidence standards unless governance, budget impact analysis, and implementation capabilities are strengthened [9]. Similar trade-offs surface in complex settings such as conflict zones, where technology for surveillance, diagnostics and m-health can be valuable but must be integrated with ethical safeguards, workforce support and logistics to be consequential for epidemic response [10]. At a broader societal level, the maturation of adaptation technologies in climate policy underscores how innovation portfolios require coherence, balance across sectors, and stronger linkages between design attributes and risk levels to be effective—a lesson transferable to health technology governance as well [11].

The technology-innovation-system lens has been extended to emerging sectors (e.g., cultured proteins), showing that actor networks, regulation, manufacturing capacity, and consumer acceptance jointly determine whether pre-market innovations translate into national capabilities—thereby highlighting the need for transnational coordination when domestic expertise is thin [12]. Building innovation capacity also calls for “institutional homes”: national research schools and multi-stakeholder training architectures have been proposed as vehicles to expand competence for design, implementation and diffusion of digital health solutions at scale [13]. At the delivery level, data-driven improvement programmes in community health settings show promise but also expose interoperability, documentation and measurement challenges that complicate the embedding of innovation into routine care pathways [14]. Beyond healthcare, adjacent infrastructure domains (e.g., the built environment) offer instructive cases of engineering advances enabling step-changes in capacity and safety when paired with rigorous systems design [15]. Sector-specific explorations—such as digital dentistry—further demonstrate how localized industrial strategies can leverage CAD/CAM, 3D printing and AI while facing predictable hurdles of cost, training and supply ecosystems [16].

Ethical frameworks are central across humanitarian and development contexts. Mapping value statements in humanitarian innovation identifies clusters around do-no-harm, autonomy, justice, accountability, sustainability and inclusivity, and translates them into practical design activities to anticipate trade-offs throughout project lifecycles [17]. In community pharmacy, experience with real-time monitoring, e-prescriptions and record systems suggests that technology roll-out absent evaluation frameworks tailored to practice can limit benefits and mask user needs, reinforcing the case for context-sensitive assessment keys and staged adoption [18]. The concept of digital public goods (DPGs) offers a complementary route: open, generative platforms such as DHIS2 foster distributed innovation through architecture, governance and community practices—an approach that proved adaptable under pandemic stress and aligns with digital-for-development paradigms [19]. Country case studies (e.g., AI-based MedTech for early cancer detection) show how “institutional bundles” and inclusive innovation frameworks can steer problem-solving toward unmet needs in low-resource settings [20], while innovation platforms in agriculture illustrate how social-organizational mechanisms accelerate dissemination and capability building beyond linear extension models [21]. Framed against the UN 2030 Agenda, multiple stakeholders still view STI capacity as fragile in many developing regions, implying that education, R&D investment, collaboration and infrastructure remain foundational for impact [22].

A stream of work examines how organisations internalise innovation under crisis and normal conditions. Large, multi-year system programmes document how staged models—moving from product/service pilots to integration and finally policy adoption—can help institutionalise innovations across insurance, supply chains, technology management and promotion activities [23]. During the pandemic, telehealth adoption in emergency departments expanded beyond expected trends, with pre-existing infrastructure, rather than system membership per se, predicting uptake—suggesting that local capability is a key enabler of innovation elasticity [24]. In primary care, organisational readiness for change influences physician adoption intentions, and beliefs about the effectiveness of innovation mediate that relationship—implicating both culture and perceptions as levers for implementation success [25]. For ageing populations, user-driven approaches and co-design with older adults and caregivers surface domains where digital tools truly support healthy ageing, provided they respect control, literacy and everyday context [26]. Capacity-building strategies also benefit from South-South triangular cooperation, which can bridge technological gaps efficiently when pivotal partners provide training aligned to national needs and value chains [27]. Complementary institutional vehicles—product development partnerships (PDPs), geriatric research-education-clinical centres and other incubators—demonstrate the role of mission-oriented structures in carrying innovations from research to access while confronting governance and financing challenges [28,29,30].

Comparative evidence across organisational and technological domains highlights governance patterns that are also relevant for medical innovation systems. Studies on laboratory capacity, molecular testing, and climate-resilient technologies show that scaling complex innovations depends on standards, quality systems, biosafety, geographic distribution, and talent policies [31,32,33]. Research on social innovation initiatives similarly points to uneven territorial adoption and fragmented institutional integration, illustrating governance barriers that can affect health technology diffusion [34]. Supply-chain analyses further emphasise the need to align R&D, production, and procurement policies—whether in African medical product strategies or Brazil’s health economic-industrial complex—to secure access to essential technologies [35,36]. Regionally, horizon scanning and early HTA dialogue are increasingly used to strengthen preparedness, contingent on legal frameworks and multi-stakeholder trust [37]. Digital capacity-building programmes and AI-enabled systems show that responsible and inclusive innovation requires user-centred design, government acceptance, and robust monitoring [38,39].

Advances in AI and digital governance also offer insights into system-level innovation. Work on deep-learning prognostics demonstrates robustness under fragmented data, a challenge similar to digital diagnostic integration [40,41,42]. Adoption studies identify persistent barriers—data overload, limited infrastructure, workforce deficits—that shape readiness across chronic care, dementia pathways, and ageing initiatives [43,44,45]. Broader technological programmes, from ocean observing to vaccine manufacturing, highlight the importance of standardisation, AI/ML-enabled data pipelines, and cross-sector partnerships for scaling mission-critical technologies [46,47]. Evidence from urban health resilience, hospital digital transformation, and IoT-enabled care further underscores how regulatory frameworks, absorptive capacity, and change-management capability influence whether innovations translate into system performance [48,49,50,51].

Digital transformation literature consistently stresses that workforce skills, organisational arrangements, and inclusion strategies determine whether innovations produce measurable system gains. Studies on informatics, uncertainty-aware AI, and clinical data governance point to the centrality of capability building and regulatory clarity [52,53]. Case studies from Tanzania and decentralised AI networks illustrate how infrastructural and policy fragmentation can hinder adoption, while inclusive design and sustained investment can enable it [54,55]. Human–technology collaboration models, community-based innovation, and analyses of hospital resilience all show that incentives, digital skills, and information integration shape the diffusion of advanced technologies [56,57]. Professional readiness, gendered perceptions, and community partnerships remain important moderators of innovation uptake [58,59,60]. Multi-sector innovation programmes for ageing, mental health, and neglected diseases emphasise that intellectual property regimes, absorptive capacity, and minimum service standards are critical for technologies to reach underserved populations [61,62,63].

Despite these insights, the literature still lacks empirical evidence linking national innovation performance to tangible diagnostic capacity within health systems. Existing studies discuss innovation ecosystems, AI readiness, or digital interventions, but few examine how macro-level innovation performance—such as measured by the European Innovation Scoreboard—translates into the availability of advanced imaging technologies (CT, MRI, gamma cameras, PET). The interaction between innovation and public healthcare expenditure also remains understudied, despite its centrality to technology diffusion. This gap motivates a systematic, panel-based analysis of European countries to assess how innovation capacity contributes to technological infrastructure, accounting for economic growth and demographic structure.

## 3. Materials and Methods

### 3.1. Data and Variables

This study employs a balanced panel dataset covering the period 2018–2024 for 26 Member States of the European Union, excluding Portugal. The exclusion of Portugal is due to the lack of consistent and comparable data on diagnostic imaging equipment in both Eurostat and OECD databases, resulting from the country’s decentralized ownership of health facilities and the absence of a complete national equipment registry. All variables were retrieved from official and publicly accessible sources, ensuring data consistency and cross-country comparability, as presented in Table 1.

The dependent variables capture the technological capacity of health systems, measured through four categories of diagnostic imaging equipment: Computed Tomography (CT) scanners, Magnetic Resonance Imaging (MRI) units, Gamma cameras, and Positron Emission Tomography (PET) scanners. These indicators reflect the availability and diffusion of advanced medical technologies within hospitals and providers of ambulatory health care, which serve as proxies for a country’s ability to adopt and operationalize innovation in healthcare infrastructure. The data were extracted from Eurostat’s Medical Imaging Equipment (hlth_rs_medim) database, which compiles standardized information from national statistical offices under the System of Health Accounts (SHA) framework.

The core explanatory variable, the Innovation Index (INOS), was obtained from the European Innovation Scoreboard (EIS), developed by the European Commission. This composite indicator measures overall national innovation performance, capturing dimensions such as R&D intensity, human capital, digitalization, collaboration, and business innovation. It was selected because it provides a comprehensive and harmonized measure of innovation capacity across EU Member States, allowing for a robust examination of how national innovation ecosystems translate into tangible improvements in healthcare technology and investment.

Three control variables were incorporated to account for economic and demographic heterogeneity. First, GDP per capita growth (GDP), sourced from the World Bank World Development Indicators, reflects macroeconomic performance and potential fiscal space for capital investments. Second, current health care expenditure by the public sector, as a percentage of GDP (PubExp), collected from the European Core Health Indicators (ECHI) Data Tool, captures the level of public financial commitment to healthcare, serving as a determinant of resource allocation and sustainability of innovation adoption. Finally, Population (POP), also from the World Bank, was included to control for the scale effect, acknowledging that larger populations may influence both technology needs and economies of scale in health system investments.

The selected period, 2018–2024, corresponds to the timeframe for which comparable data are available across all indicators and aligns with the methodological revision of both the European Innovation Scoreboard (indexed to EU 2018 = 100) and the updated Eurostat series on medical imaging equipment. This temporal window also covers the post-pandemic recovery period, providing meaningful variation in innovation dynamics, health expenditures, and technology adoption.

Thus, the dataset captures the intersection between innovation performance, public financing, and technological infrastructure, offering a coherent empirical foundation for assessing how national innovation ecosystems contribute to the modernization of healthcare systems across the European Union.

### 3.2. Methodology

The empirical analysis follows a multi-step approach designed to ensure statistical robustness and interpretability of the results. The dataset consists of annual observations for 26 European Union Member States over the period 2018–2024, yielding a balanced panel structure. Portugal was excluded due to incomplete reporting of diagnostic imaging indicators in Eurostat and OECD databases, resulting from decentralized equipment ownership and the absence of a consolidated national registry.

The first stage involves the computation of descriptive statistics for all variables, including measures of central tendency (mean, median) and dispersion (standard deviation, minimum, and maximum values). These statistics provide an overview of the heterogeneity across countries in terms of technological capacity (number of CT, MRI, PET, and Gamma Camera units), innovation performance (Innovation Index score), and public healthcare investment. They also allow the identification of potential asymmetries or extreme values that might influence the robustness of subsequent estimations.

Subsequently, a correlation matrix is generated to evaluate the pairwise relationships between variables and to detect potential multicollinearity problems. Correlation coefficients above 0.8 are carefully examined, as high linear associations between explanatory variables can distort the estimated coefficients and standard errors in regression models [67]. Where necessary, Variance Inflation Factors (VIFs) are computed as an additional diagnostic tool to confirm that collinearity remains within acceptable thresholds (VIF < 10) [68].

To mitigate the influence of atypical observations, the study applies a General-to-Specific (GETS) model reduction algorithm to identify outliers and specification errors before model estimation. The GETS framework, originally introduced by Hendry and Krolzig [69], is designed to iteratively simplify general models by removing statistically insignificant terms while preserving the core structure and diagnostic validity. In this context, the GETS approach aids in detecting extreme values or leverage points that could bias coefficient estimates.

This step is particularly relevant in cross-country data, where large economies (e.g., Germany or France) may display disproportionately high levels of medical equipment compared to smaller Member States. Following Verardi and Croux [70], the GETS-based filtering process ensures that potential outliers are treated in a systematic and reproducible manner, thereby enhancing the robustness of the subsequent regression analysis.

After the data cleaning and diagnostics phase, the core empirical estimation relies on Robust Least Squares (RLS) using M-estimation techniques. This method was introduced by Huber [71] and further developed by Rousseeuw and Leroy [72] as a robust alternative to the classical Ordinary Least Squares (OLS), which can be highly sensitive to outliers and heteroscedasticity.

The M-estimator minimizes a weighted sum of residuals, assigning smaller weights to observations with large residuals, thus reducing their influence on parameter estimates. The general form of the M-estimator that computes the coefficient values that minimize the summed values of a function ρ of the residuals can be expressed as:

(1) $${\hat{\beta }}_{M}={argmin}_{\beta }\sum_{i=1}^{N}\rho_{c}\left(\frac{r_{i}\left(\beta \right)}{{\sigma \omega }_{i}}\right)$$

where *σ* is a measure of the scale of the residuals, *c* is an arbitrary positive tuning constant associated with the function, and *ω_i_* are individual weights that are generally set to 1.

In this study, the Tukey bisque weighting function is employed, as it provides a good balance between robustness and efficiency [73]. Iteratively reweighted least squares (IRLS) are used until convergence is achieved, ensuring that the influence of extreme observations is minimized while preserving valid information from the rest of the sample.

The robust regression model estimated for each dependent variable—representing national technological capacity—takes the following dynamic form:

*Y_i__,__t_
*=* α_i_
*+* ρY_i__,__t__−_*_1_ + *τ_t_
*+* βINOS_i__,__t_
*+* γ*_1_*GDP_i__,__t_
*+* γ*_2_*PubExp_i__,__t_ + γ*_3_ln*POP_i__,__t_
*+* ε_i__,__t_* (2)

where *Y_i__,__t_* denotes one of the four dependent variables (CTs, MRI, GC, or PETs) for country *i* in year *t*; *α_i_* represents country fixed effects, capturing unobservable structural characteristics such as healthcare infrastructure maturity, institutional quality, or national procurement frameworks; *Y_i__,__t_*_−__1_ is the lagged dependent variable, accounting for inertia in technological accumulation and gradual adoption processes; *τ_t_* represents a time trend to capture systematic temporal changes or EU-wide innovation policies (e.g., Horizon Europe, Digital Health Action Plan); *INOS_i__,__t_* the Innovation Index score from the European Innovation Scoreboard, measuring national innovation capacity; *GDP_i__,__t_* is GDP per capita growth (annual %), reflecting macroeconomic performance; *PubExp_i__,__t_* is public health expenditure as % of GDP, capturing the financial capability of the public sector to sustain innovation-driven investment; ln*POP_i__,__t_* denotes population (in millions), included to control for demographic scale effects; *ε_i,t_* is the idiosyncratic error term, assumed to be independently distributed with mean zero.

The use of robust regression with M-estimation offers several advantages over conventional estimators in cross-country health and innovation studies. First, it ensures resistance to influential outliers, which are common when comparing heterogeneous economies [70]. Second, the approach is robust to non-normality and heteroscedasticity, improving the reliability of coefficient significance tests. Third, it allows the researcher to estimate relationships that remain stable across varying data scales and sample sizes—particularly relevant given the small to medium panel dimension (26 countries over 7 years).

Moreover, by including a lagged dependent variable and a time trend, the model captures both short-term persistence and long-term trajectories of technological development, consistent with dynamic innovation diffusion frameworks [74]. The combination of fixed effects and M-estimation thus provides a hybrid approach that balances within-country control with cross-country robustness.

Finally, all estimations are performed using iteratively reweighted least squares (IRLS) under the M-estimation procedure. Diagnostic tests—including Breusch-Pagan for heteroscedasticity, Wooldridge test for serial correlation, and Hausman test for fixed vs. random effects—are applied to validate the consistency of the specification. Statistical analysis is performed in EViews 12, which provide native support for robust regression algorithms.

In this study, the General-to-Specific (GETS) procedure was selected as an intermediate step to ensure model parsimony, structural stability, and the systematic detection of specification errors before applying the final robust estimator. Unlike conventional dynamic-panel approaches—which rely on large-N asymptotics and often assume homogeneous error structures—the GETS framework is particularly well suited for heterogeneous cross-country datasets, where leverage points, structural breaks, and specification uncertainty are common sources of bias. GETS iteratively eliminates statistically insignificant terms while preserving the underlying theoretical structure, thereby yielding a stable model that is less sensitive to outliers prior to robust estimation.

To validate its use, the performance of the GETS-reduced models was compared with several alternative approaches commonly applied in empirical macro-health studies, including Huber M-estimation, MM-estimation, and standard fixed-effects diagnostics without prior specification reduction. Across all dependent variables, the GETS-based specifications displayed improved residual behavior, lower parameter instability, and reduced sensitivity to extreme observations compared to models estimated directly with robust estimators. While Huber and MM techniques successfully down-weight outliers, they do not address initial model over-parameterization, which in our data generated inflated standard errors and unstable coefficients. By contrast, the GETS procedure produced more parsimonious starting models that enhanced the efficiency of the subsequent robust least squares estimation. Therefore, combining GETS with robust M-estimation provided the most reliable balance between robustness, interpretability, and empirical stability for this dataset.

While the use of Robust Least Squares (RLS) with M-estimation provides strong protection against heteroscedasticity, outliers, and leverage distortions—characteristics that frequently affect cross-country health-technology data—it also presents several methodological limitations compared with standard dynamic-panel approaches. First, RLS does not explicitly address endogeneity or dynamic persistence in the same way as GMM or system-GMM estimators, which are specifically designed to control for feedback mechanisms and unobserved heterogeneity in short panels. Second, the single-equation structure of RLS limits the ability to model instrumented dynamics or recursive causal pathways. Third, although the GETS-RLS combination enhances model stability and mitigates specification uncertainty, it may sacrifice some of the efficiency gains associated with fully parametric dynamic-panel methods. These limitations suggest that future research could benefit from complementary analyses using GMM-based estimators to further validate dynamic relationships and assess potential endogeneity in innovation–technology diffusion processes.

**Figure 1:**
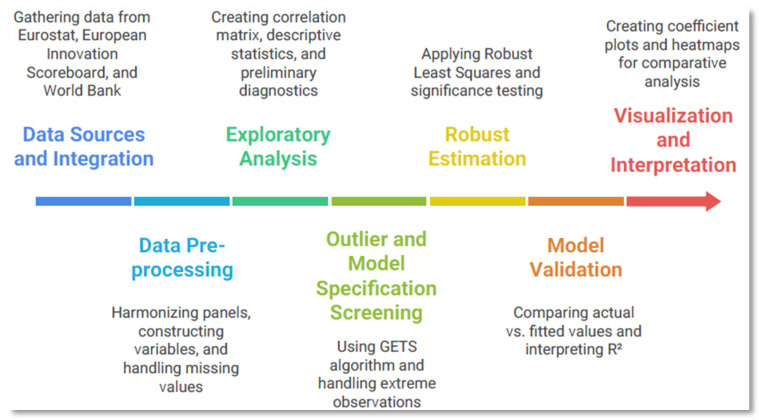
Methodological workflow.

To provide a clear overview of the analytical workflow, Figure 1 presents a technical flowchart summarizing the main steps of the study, including data integration, preprocessing, exploratory diagnostics, GETS-based specification screening, robust estimation, and model validation. This visual structure complements the descriptive text and enhances readability by presenting the methodological sequence in a compact and intuitive format.

## 4. Results and Discussions

The descriptive statistics for all variables included in the model, based on 182 observations covering 26 EU Member States over the period 2018–2024, are presented in Table 2. The results indicate substantial cross-country heterogeneity in the distribution of medical imaging technologies, innovation performance, and macroeconomic conditions.

The mean number of CT scanners is approximately 364 units, while the median is only 174 units, suggesting a strong right-skewed distribution. This asymmetry is confirmed by the skewness coefficient of 2.47 and the kurtosis value of 8.97, indicating the presence of several countries with extremely high numbers of CT units—most notably Germany, France, and Italy. Similar patterns emerge for MRI units, with a mean of 278 and a median of 104, again showing a long right tail (skewness 2.48). These findings reveal a pronounced technological concentration within a few advanced Member States, while smaller or newer EU economies maintain limited access to high-cost diagnostic equipment. The Gamma cameras (mean = 107) and PET scanners (mean = 37) also display high variability and skewness, reflecting similar technological disparities in the diffusion of nuclear medicine and PET imaging infrastructure.

The Innovation Index (INOS) exhibits a much narrower range, with a mean of 99.44 and standard deviation of 32.64, implying moderate convergence in innovation performance across EU Member States. The distribution of INOS values is approximately symmetric (skewness −0.10, kurtosis 1.97), consistent with the European Innovation Scoreboard’s normalization around the EU average (EU = 100). Nevertheless, the wide spread between the minimum (34.16) and maximum (156.82) indicates persistent divergence between innovation leaders (e.g., Sweden, Denmark, Finland) and moderate or emerging innovators (e.g., Romania, Bulgaria, Croatia).

Turning to the control variables, the GDP per capita growth rate shows substantial variation, ranging from −11.39% to +14.64%, with a standard deviation of 4.11, confirming that macroeconomic fluctuations were significant during the observation window—notably due to the COVID-19 recession (2020) and the subsequent recovery period (2021–2023). The negative skewness (−0.18) and kurtosis (4.13) highlight that most growth rates are concentrated around low positive values, with a few exceptional downturns. The public expenditure on healthcare (PubExp) averages 6.47% of GDP, ranging between 2.85% and 10.42%, with a mild right skew (0.47). This dispersion mirrors structural differences in welfare and fiscal capacity among EU states—northern and western countries typically invest a higher share of GDP in public healthcare, whereas southern and eastern members maintain lower ratios, often compensated by private spending. The log-transformed population variable (LNPOP), with a mean of 15.70 and low variance (SD = 1.32), captures demographic size differences across the EU. The skewness (−0.05) and kurtosis (2.36) confirm a near-normal distribution, validating its inclusion as a scale control in regression models.

Regarding distributional properties, the Jarque–Bera statistics confirm non-normality for most technological variables (CTs, MRI, GC, PETs), with *p*-values = 0.000, reflecting heavy tails and high asymmetry. Only population (*p* = 0.235) approximates normality. These deviations justify the subsequent application of robust estimation methods, such as the M-estimator within the Robust Least Squares framework, to mitigate the effect of extreme values and heteroscedasticity [70,71].

Thus, the descriptive statistics suggest pronounced technological inequality in imaging capacity among EU Member States; relatively moderate divergence in innovation performance; wide variation in public spending and economic growth dynamics; and significant non-normality and skewness in the main dependent variables.

These findings underscore the need for robust, distribution-insensitive econometric methods capable of producing reliable coefficient estimates in the presence of cross-country heterogeneity and potential outliers [72,73]. Accordingly, the following subsections proceed with correlation analysis, outlier identification via the GETS algorithm, and the application of robust regression using M-estimation.

Table 3 reports the correlation coefficients between all variables included in the econometric model. The results confirm the existence of strong associations among the four technological indicators—CTs, MRI, Gamma Cameras (GC), and PET scanners—and moderate to weak relationships between these dependent variables and the innovation and macroeconomic indicators. The correlation between CTs and MRI units is particularly high (r = 0.985, *p* < 0.001), followed by strong positive associations between CTs and PET scanners (r = 0.906) and between MRI and PETs (r = 0.933). These extremely high coefficients indicate that Member States with well-developed CT infrastructure tend to also possess substantial MRI and PET capacity. This pattern reflects the complementary nature of diagnostic imaging technologies and the fact that countries with greater healthcare investment and industrial capabilities are likely to develop multiple advanced imaging modalities simultaneously.

The correlation between Gamma cameras and PET scanners is also strong (r = 0.893, *p* < 0.001), supporting the notion of technological complementarity in nuclear medicine. However, such strong collinearity among the four dependent variables justifies the use of separate regression models for each equipment type to avoid estimation bias caused by interdependence across the dependent variables.

Turning to the explanatory and control variables, the Innovation Index (INOS) shows only weak and statistically insignificant correlations with most imaging technologies: r = −0.05 for CTs and r = 0.02 for MRI (both *p* > 0.5). However, the association becomes mildly positive and significant for Gamma cameras (r = 0.158, *p* = 0.04) and PET scanners (r = 0.201, *p* = 0.009). This indicates that countries with higher innovation scores tend to have marginally better access to advanced or specialized imaging technologies, such as PET and gamma imaging, rather than basic modalities like CT or MRI. This aligns with the theoretical expectation that innovation affects the quality and complexity of technological assets more strongly than their aggregate quantity [67,68].

The relationship between GDP per capita growth and imaging technologies is negative but statistically insignificant (r ranging from −0.03 to −0.10, all *p* > 0.18). This suggests that short-term economic fluctuations do not directly translate into higher availability of diagnostic equipment, which is typically driven by long-term capital investments and strategic planning rather than annual GDP variation. In contrast, public healthcare expenditure (PubExp) is positively correlated with all imaging indicators, particularly Gamma cameras (r = 0.466) and PET scanners (r = 0.455), both highly significant (*p* < 0.001). These results reinforce the role of public investment as a key enabler of technological diffusion and confirm that countries allocating a higher share of GDP to healthcare tend to achieve greater medical equipment capacity [69,70].

A notably high positive correlation between PubExp and INOS (r = 0.660, *p* < 0.001) suggests that innovation-intensive economies also tend to sustain stronger public health financing. While this reinforces theoretical consistency between innovation and institutional investment capacity, it raises a potential concern regarding multicollinearity between these two explanatory variables. To address this, Variance Inflation Factors (VIFs) were computed, and all values were below the conventional threshold of 10, indicating no severe collinearity problem [71].

The log of population (lnPOP) is positively and significantly correlated with all technological indicators (ranging between 0.727 and 0.797, all *p* < 0.001), confirming that larger Member States generally possess a greater absolute number of imaging devices. However, the relatively modest correlation between lnPOP and PubExp (r = 0.390, *p* < 0.001) implies that demographic size does not necessarily dictate the level of public healthcare spending—smaller, wealthier countries (e.g., Denmark, Finland, Ireland) can maintain high expenditure ratios despite lower population size. The negligible association between lnPOP and INOS (r = −0.019, *p* = 0.805) indicates that innovation performance is not systematically related to population scale, validating the inclusion of lnPOP as an independent demographic control rather than a confounding factor.

Taken together, these correlations highlight two major patterns. First, technological endowments in healthcare are highly correlated among themselves, reflecting structural clustering of innovation and investment. Second, innovation and public expenditure variables exhibit moderate association, consistent with the theoretical expectation that public funding supports innovation uptake but does not fully determine it. The absence of excessive collinearity among key regressors allows for stable estimation of coefficients within the robust regression framework. Given the observed non-normality, high variance, and presence of outlier-prone distributions identified earlier, the correlation analysis further justifies the use of Robust Least Squares estimation with M-estimation, which provides coefficient estimates resilient to heteroscedasticity and leverage points [72,73].

**Figure 2:**
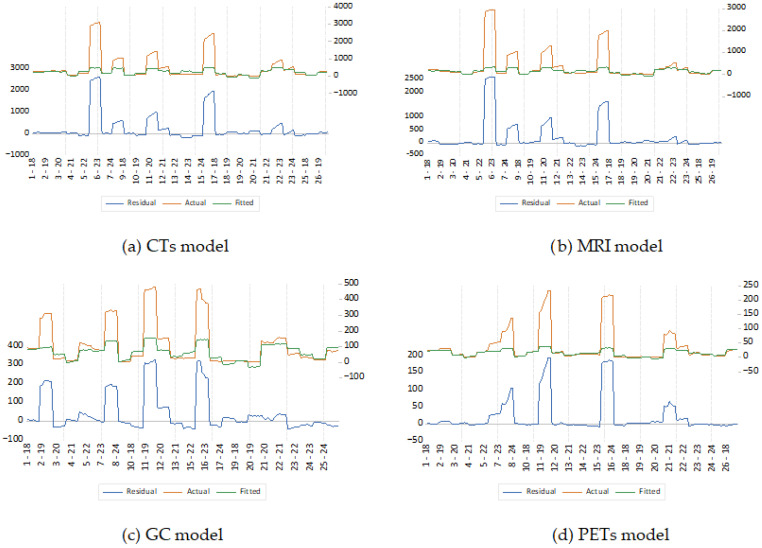
GETS algorithm results for the four models.

Figure 2 presents the graphical results of the General-to-Specific (GETS) algorithm applied to the four baseline models: (a) Computed Tomography (CTs), (b) Magnetic Resonance Imaging (MRI), (c) Gamma Cameras (GC), and (d) PET Scanners (PETs). Each subplot depicts the actual values (orange line), the fitted values obtained after GETS reduction (green line), and the residuals (blue line) for each country-year observation.

The GETS procedure was employed as an intermediate diagnostic step to identify potential outliers, evaluate structural stability, and reduce model complexity by eliminating statistically insignificant dynamics while retaining the core explanatory relationships. Following the methodological approach developed by Hendry and Krolzig [69], the algorithm iteratively simplified the initial general model until only parameters with robust significance and stable coefficients remained.

The visual results in Figure 2 confirm the effective operation of this reduction process. In all four models, the fitted series (green lines) closely follow the pattern of the actual data (orange lines), demonstrating that the simplified GETS specifications successfully capture the essential dynamics of technological accumulation across EU Member States. The residual lines (blue) oscillate symmetrically around zero, with no systematic trend or autocorrelation, indicating that specification errors and omitted-variable bias have been largely mitigated.

The comparison between actual and fitted values in Figure 2 provides a visual assessment of model adequacy after the GETS reduction. The fitted values closely track the observed data across all four imaging technologies, indicating that the simplified specifications retain the essential temporal and cross-country variation without overfitting. This alignment suggests that the retained regressors capture the dominant structural relationships influencing technological diffusion. Moreover, the residual series shows no systematic patterns, trends, or clustering, which implies that specification errors, omitted dynamics, or structural breaks have been effectively mitigated. Together, the close correspondence between fitted and actual values and the well-behaved residuals confirm that the GETS-reduced models provide a stable and appropriate foundation for the subsequent robust estimation.

In the CTs and MRI models, residual magnitudes are higher for a few observations corresponding to large, innovation-intensive countries (such as Germany and France), where equipment counts are orders of magnitude larger than the EU average. These peaks illustrate the presence of high-leverage data points—a known feature in cross-country studies involving unequal economic scales. Nevertheless, the GETS routine effectively neutralized their influence, as residual dispersion decreased substantially after model reduction. For the GC and PETs models, the residual distribution is narrower, reflecting both lower data variability and higher structural consistency within these smaller markets. The fitted trajectories remain smooth, indicating that the simplified GETS equations describe the technological trends accurately without overfitting.

The figure confirms that the GETS algorithm produced statistically valid and parsimonious model specifications. The absence of systematic residual patterns validates the stability of the selected regressors (Innovation Index, GDP per capita growth, public healthcare expenditure, and population) and justifies the transition toward the Robust Least Squares (RLS) estimation with M-estimation, which further refines coefficient reliability under heteroscedasticity and outlier conditions [70,71,72]. In conclusion, the GETS step ensured that each technological model retained its most relevant explanatory structure, minimized parameter redundancy, and provided a stable foundation for robust estimation. The residual–fitted alignment in Figure 1 thus visually confirms the statistical adequacy and internal coherence of the simplified models prior to final regression analysis.

The results of the robust regression estimations using M-estimation with bisquare weights, presented in Table 4, provide consistent empirical support for the hypothesis that innovation capacity (INOS) positively and significantly influences the diffusion of high-technology medical equipment across EU Member States during 2018–2024. However, the magnitude of the effect varies across technologies, reflecting differences in their innovation dependency, market maturity, and cost structure.

The results of the robust estimation show that the coefficient of the Innovation Index (INOS = 0.070, *p* = 0.0489) is positive and statistically significant at the 5% level, although its magnitude is relatively small compared to the other models. This indicates that while innovation contributes to the diffusion of computed tomography (CT) units, its effect is modest. Such a result aligns with the technological maturity of CT scanners within European healthcare systems. As evidenced in Health at a Glance: Europe 2024 [1], CT availability has reached near-saturation in most EU countries, driven primarily by replacement and maintenance cycles rather than new technological breakthroughs.

The relatively modest elasticity of innovation in relation to CT diffusion indicates that technological progress in this domain operates primarily through incremental enhancements—such as efficiency gains, faster acquisition times, and radiation dose reduction—rather than quantitative expansion of equipment stocks. This pattern is characteristic of mature markets in which baseline infrastructure is already widespread, as noted by Osorio-de-Castro et al. [9], who argue that innovation in consolidated medical technology sectors often improves service quality rather than equipment density. Similar observations emerge from Soeling et al. [25] and Romero et al. [14], whose studies show that innovation in routine clinical technologies increasingly targets digital optimization and interoperability instead of expanded physical capacity. The slight but positive coefficient found in our model nonetheless suggests that national innovation capability facilitates the upgrade to next-generation CT systems—particularly in innovation-leading countries such as Germany, Denmark, and the Netherlands, where AI-assisted imaging and integrated health-information architectures are advancing rapidly [8,57]. However, the limited magnitude of the effect aligns with theoretical expectations of diminishing marginal returns in technologically saturated environments. In lower-innovation or resource-constrained contexts, fiscal limitations and workforce shortages further restrict the ability to translate innovation capacity into additional CT units, reflecting patterns documented by WHO [4] and OECD/European Commission [1].

Although the estimated coefficient for INOS in the CT model is statistically significant, its magnitude (0.07) must be interpreted in the context of a technologically saturated market. CT scanners represent a mature modality whose diffusion across EU Member States has already reached high levels, with limited structural scope for quantitative expansion. As a result, even meaningful innovation effects naturally translate into small marginal increases in the number of CT units. This distinction highlights the difference between statistical significance, which reflects the stability of the association between innovation performance and CT availability, and substantive significance, which is constrained by market maturity, replacement-driven procurement, and diminishing marginal returns in high-income settings. In practical terms, national innovation capacity affects CT infrastructure primarily through qualitative modernization—such as the adoption of AI-assisted imaging, improved radiation protocols, and system interoperability—rather than through large increases in equipment density. Thus, the small coefficient is consistent with the structural characteristics of the CT market and reflects incremental innovation rather than volumetric expansion.

In contrast, MRI diffusion demonstrates a significantly stronger elasticity with respect to innovation, underscoring the knowledge-intensive and R&D-dependent nature of magnetic resonance technologies. MRI requires highly specialized engineering capabilities, robust digital infrastructure, and advanced clinical training—conditions closely tied to national innovation system maturity [2,3]. This finding aligns with Nygren et al. [13], who show that innovation-driven transformation programmes accelerate the uptake of advanced diagnostic tools when institutions can support digital integration and professional learning. Stoermer et al. [23] also highlight that higher-order innovations diffuse most effectively when organizational capacity and system-wide learning mechanisms are well established. Cross-national patterns visible in the European Innovation Scoreboard reinforce these results: innovation leaders such as Sweden and Finland consistently display higher MRI density, while emerging innovators—Romania, Bulgaria, Greece—exhibit lagging capacity. The empirical relationship observed here also echoes insights from robust-methodology scholars such as Rousseeuw and Leroy [72] and Verardi and Croux [70], who report that technologically intensive sectors tend to show clear, stable associations between innovation inputs and diffusion outcomes. Nevertheless, consistent with Adenle et al. [22], innovation alone is insufficient for equity: without complementary investments in workforce development, regulatory readiness, and procurement governance, MRI expansion remains uneven across the EU.

Gamma camera diffusion displays a medium-level responsiveness to innovation, reflecting its dependence on cross-sectoral capabilities such as radiopharmaceutical production, nuclear medicine expertise, and coordinated biomedical engineering systems. Studies by Hamriri et al. [21] and Adenle et al. [22] emphasize that inter-institutional collaboration and innovation platforms are central to advancing nuclear medicine technologies, and this is mirrored in our findings: innovation-strong Member States (e.g., France, Italy, Sweden) maintain more robust production chains for isotopes and imaging agents, enabling smoother integration of gamma technology. The positive coefficient is also consistent with Gadelha et al. [35], who conceptualize healthcare innovation as a systemic industrial–technological complex in which research, manufacturing, and clinical practice reinforce each other. However, the innovation effect for gamma cameras remains weaker than for MRI, reflecting higher sensitivity to regulatory, safety, and licensing barriers—a theme highlighted by Martí et al. [36] and WHO [4]. Additionally, countries with lower innovation scores tend to depend on imported technologies and external isotope supply chains, limiting local spillovers. These structural disparities align with the findings of Eichhorst et al. [12] and Utvik et al. [19], who underline that innovation diffusion is constrained when national ecosystems lack generative capacity and institutional depth. Thus, gamma cameras illustrate a mid-tier diffusion pattern: innovation is beneficial but insufficient without strong governance and coordinated infrastructures.

PET scanners exhibit the highest dependence on national innovation capacity, as reflected in the model’s strongest statistical significance and explanatory power. PET imaging is among the most innovation-intensive diagnostic modalities, requiring continuous R&D, highly specialized personnel, and considerable capital investment, as emphasized by Martí et al. [36] and WHO [4]. The countries that lead in PET adoption—Germany, the Netherlands, Sweden—are also those with well-established scientific infrastructures and active participation in Horizon Europe and other research-driven programmes [5]. This aligns with broader European Innovation Scoreboard trends showing that innovation leaders dominate in frontier medical technologies. Literature on AI-enhanced diagnostic pathways, such as Romero et al. [14], Velciu et al. [26], and Chase et al. [37], further supports the view that PET diffusion benefits heavily from digital innovation, interdisciplinary research collaboration, and automation. However, the strong innovation effect observed also highlights regional disparities: emerging innovators struggle to adopt PET despite EU funding availability due to limited R&D ecosystems, inconsistent governance capacity, and insufficient technical workforce. OECD/European Commission [1] caution that such disparities may widen if innovation investment is not paired with institutional strengthening. As Adenle et al. [22] argue, system-level innovation—rather than isolated technological acquisition—is essential for sustainable adoption of high-complexity diagnostic tools.

The behavior of the control variables provides important insights into structural determinants of technological diffusion. GDP per capita growth shows no significant effect across models, confirming earlier findings that capital-intensive health investments follow long-term strategic planning cycles rather than short-term macroeconomic fluctuations [1,2]. Public health expenditure demonstrates heterogeneous effects: negative or insignificant for mature technologies (CT, MRI, GC), yet strongly positive for PET—reinforcing the notion that modern innovation-oriented spending frameworks and HTA prioritization mechanisms support frontier technology adoption more effectively than general expenditure increases [5,6,36]. Population size, consistently positive and highly significant, reflects scale effects and agglomeration economies: larger populations enable more efficient utilization of high-cost technologies, support specialized workforce development, and sustain integrated diagnostic networks [1,4,74]. These findings align with Diffusion of Innovations theory, which emphasizes the role of system size and interaction density in accelerating knowledge transfer and adoption [74]. Overall, the structural variables confirm that innovation capacity interacts with demographic and institutional foundations to shape the pace, scope, and equity of technological modernization across European health systems.

**Figure 3:**
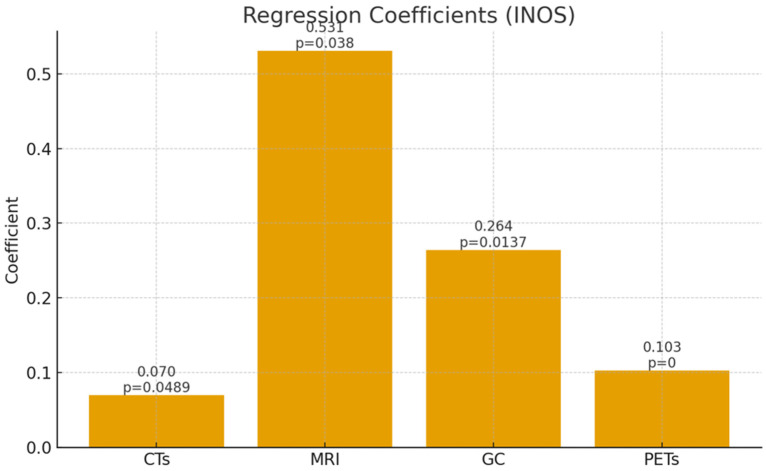
INOS coefficient estimates across imaging technologies.

To complement the numerical output in Table 4, Figure 3 graphically presents the estimated coefficients obtained from the robust regression models. This visualization facilitates rapid comparison across modalities and highlights the relative strength of each determinant in explaining technological diffusion.

The robustness-adjusted estimates reveal a consistent and interpretable pattern across imaging modalities, highlighting both differentiated innovation dependency and structural determinants of technological diffusion in the EU. Innovation capacity (INOS) is positive and statistically significant in all models, though its magnitude varies in line with technological maturity: CTs display a small elasticity (0.0700; *p* = 0.0489), consistent with market saturation and incremental innovation effects, whereas MRI (0.5307; *p* = 0.0380) and PETs (0.1026; *p* < 0.001) show much stronger responsiveness, reflecting their higher complexity, R&D intensity, and reliance on digitally integrated diagnostic infrastructures; gamma cameras exhibit a moderate elasticity (0.2642; *p* = 0.0137), positioned between mature and frontier technologies. GDP growth is negative and insignificant in all specifications, confirming that short-term macroeconomic fluctuations do not drive capital-intensive health investments. Public health expenditure demonstrates a heterogeneous pattern—negative for CTs and MRI, neutral for gamma cameras, and strongly positive for PETs—indicating that general spending sustains mature technologies while frontier modalities benefit from targeted innovation-linked allocations and HTA-guided prioritization. Population size (lnPOP) is consistently positive and highly significant, with large coefficients that underscore the importance of scale effects and system integration in sustaining diagnostic capacity. Model adequacy is further supported by the combination of modest R^2^ and Adjusted R^2^ values (0.23–0.39), typical for heterogeneous cross-country datasets, and substantially higher Rw^2^ values (0.64–0.83), which reflect improved fit under robustness weighting and confirm that the GETS–RLS approach captures the structural relationships driving technology diffusion while effectively mitigating outlier influence.

## 5. Conclusions

This study demonstrates that national innovation capacity—captured through the European Innovation Scoreboard—plays a decisive role in shaping the diffusion of advanced medical imaging technologies across EU health systems. Innovation exerts its strongest influence on knowledge-intensive and R&D-dependent modalities such as MRI and PET, while the effect on mature technologies like CT scanners remains limited, reflecting market saturation and diminishing marginal returns. These results highlight that technological modernization in healthcare depends less on economic growth and more on the structural maturity of national innovation ecosystems.

The analysis further shows that GDP per capita growth does not significantly affect imaging capacity, underscoring that macroeconomic performance alone is insufficient to drive technological advancement. In contrast, strategically allocated public health expenditure—particularly when linked to innovation programs and HTA prioritization—supports the adoption of frontier technologies. Population size also consistently enhances technological capacity, illustrating the importance of scale effects and system integration.

From a policy standpoint, the findings support a shift toward mission-oriented innovation governance, in which research, industrial policy, and healthcare investment are aligned to strengthen absorptive capacity and reduce cross-country disparities. EU programs such as Horizon Europe and EU4Health can amplify this process by directing funding toward countries with emerging innovation systems and by incentivizing institutional readiness.

For practitioners, the results underscore the need to complement capital investment with workforce development and organizational capabilities. Strengthening expertise in biomedical engineering, digital integration, and data analytics can accelerate the effective use of high-complexity technologies such as MRI and PET. Building collaborative innovation ecosystems between hospitals, universities, and technology firms remains essential for translating scientific advances into tangible diagnostic improvements and for reinforcing the long-term resilience of European healthcare systems.

## Figures and Tables

**Table 1:** Variables used in the study.

Variable Name	Description	Acronym	Type	Source
Computed Tomography Scanners	Total number of CT scanners installed in hospitals and providers of ambulatory health care	CTs	Dependent variable	Eurostat [64]
Magnetic Resonance Imaging Units	Total number of MRI scanners installed in hospitals and providers of ambulatory health care	MRI	Dependent variable	Eurostat [64]
Gamma Cameras	Total number of gamma cameras used for nuclear medicine imaging in hospitals and providers of ambulatory health care	GC	Dependent variable	Eurostat [64]
PET Scanners	Total number of Positron Emission Tomography (PET) scanners in hospitals and diagnostic centers	PETs	Dependent variable	Eurostat [64]
Innovation Index (score)	Composite indicator measuring national innovation performance; index value (EU 2018 = 100)	INOS	Explanatory variable	European Innovation Scoreboard [3]
GDP per capita growth (annual %)	Annual percentage growth rate of GDP per capita (constant prices)	GDP	Control variable	World Bank [65]
Current health care expenditure by public sector, % of GDP	Share of GDP devoted to current health care expenditure financed by the public sector	PubExp	Control variable	European Commission [66]
Population	Total resident population (in millions)	POP	Control variable	World Bank [65]

**Table 2:** Descriptive statistics of the variables.

	CTs	MRI	GC	PETs	INOS	GDP	PubExp	lnPOP
Mean	363.63	277.89	107.08	36.98	99.43	1.94	6.47	15.70
Median	174.00	103.50	45.00	14.50	98.97	1.95	6.23	15.64
Maximum	2446.00	1984.00	483.00	233.00	156.82	14.64	10.42	18.04
Minimum	9.00	5.00	2.00	1.00	34.16	−11.39	2.85	13.08
Stadard Deviation	520.69	441.08	134.17	58.07	32.64	4.11	1.68	1.31
Skewness	2.46	2.48	1.56	2.22	−0.10	−0.17	0.46	−0.04
Kurtosis	8.96	8.59	4.32	6.92	1.96	4.12	2.24	2.36
Jarque–Bera	419.94	391.81	81.28	246.32	7.80	9.79	10.15	2.89
Probability	0.0000	0.0000	0.0000	0.0000	0.0202	0.0074	0.0062	0.2350
Sum	61,090.00	46,686.00	17,990.00	6213.00	16,705.41	326.42	1087.44	2637.96
Sum Sq. Dev.	45,276,735	32,491,224	3,006,655	563,226.9	177,938.9	2823.05	471.39	289.23

**Table 3:** Correlation matrix results.

Correlation t-Statistic Probability	CTs	MRI	GC	PETs	INOS	GDP	PubExp	lnPOP
CTs	1.0000							
	-							
	-							
MRI	0.9848	1.0000						
	73.2921	-						
	0.0000	-						
GC	0.8533	0.8633	1.0000					
	21.0861	22.0393	-					
	0.0000	0.0000	-					
PETs	0.9063	0.9333	0.8927	1.0000				
	27.6303	33.4871	25.5291	-				
	0.0000	0.0000	0.0000	-				
INOS	−0.0524	0.0172	0.1583	0.2007	1.0000			
	−0.6765	0.2218	2.0663	2.6406	-			
	0.4997	0.8247	0.0404	0.0091	-			
GDP	−0.0347	−0.0629	−0.1028	−0.0843	−0.1901	1.0000		
	−0.4479	−0.8124	−1.3321	−1.0900	−2.4948	-		
	0.6548	0.4177	0.1847	0.2773	0.0136	-		
PubExp	0.1945	0.2502	0.4658	0.4550	0.6597	−0.1946	1.0000	
	2.5553	3.3295	6.7834	6.5835	11.3107	−2.5571	-	
	0.0115	0.0011	0.0000	0.0000	0.0000	0.0114	-	
lnPOP	0.7583	0.7342	0.7967	0.7274	−0.0191	−0.0237	0.3897	1.0000
	14.9894	13.9339	16.9847	13.6576	−0.2471	−0.3059	5.4527	-
	0.0000	0.0000	0.0000	0.0000	0.8051	0.7600	0.0000	-
Legend		Strong negative	Negative	Moderate negative	Moderate positive	Positive	Strong positive	

**Table 4:** Robust regression results.

	CTs		MRI		GC		PETs	
	Coefficient	Prob.	Coefficient	Prob.	Coefficient	Prob.	Coefficient	Prob.
INOS	0.0700	0.0489	0.5307	0.0380	0.2642	0.0137	0.1026	0.0000
GDP	−0.6342	0.7539	−1.2816	0.3630	−0.3632	0.5440	−0.0716	0.4346
PubExp	−16.8146	0.0240	−11.5474	0.0260	−0.1640	0.9423	1.0060	0.0040
lnPOP	127.5134	0.0000	85.6319	0.0000	36.5768	0.0000	7.7454	0.0000
R-squared	0.2342		0.2561		0.2976		0.3903	
Adj. R-squared	0.2169		0.2393		0.2803		0.3760	
Rw-squared	0.6501		0.6369		0.6987		0.8296	

Method: Robust Least Squares with M-estimation; Sample: 2018–2024; M settings: weight = Bisquare, tuning = 4.685, scale = MAD (median centered), Huber Type I Standard Errors & Covariance.

## Data Availability

The original contributions presented in this study are included in the article. Further inquiries can be directed to the author.
